# Supplementary material for: Altered Plasma microRNA Signature in Hospitalized COVID-19 Patients Requiring Oxygen Support
Source: Microorganisms. 2024 Feb 21;12(3):440. doi: 10.3390/microorganisms12030440 (PMC10972147; doi:10.3390/microorganisms12030440)
Supplement: Supplementary file 1 [file microorganisms-12-00440-s001.zip › Supplementary Figure S1.pdf]

A

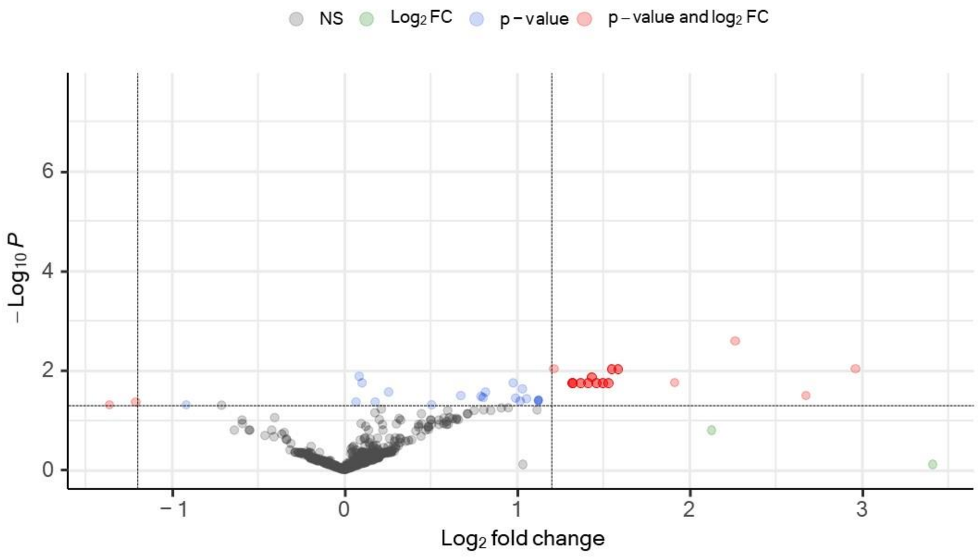

B

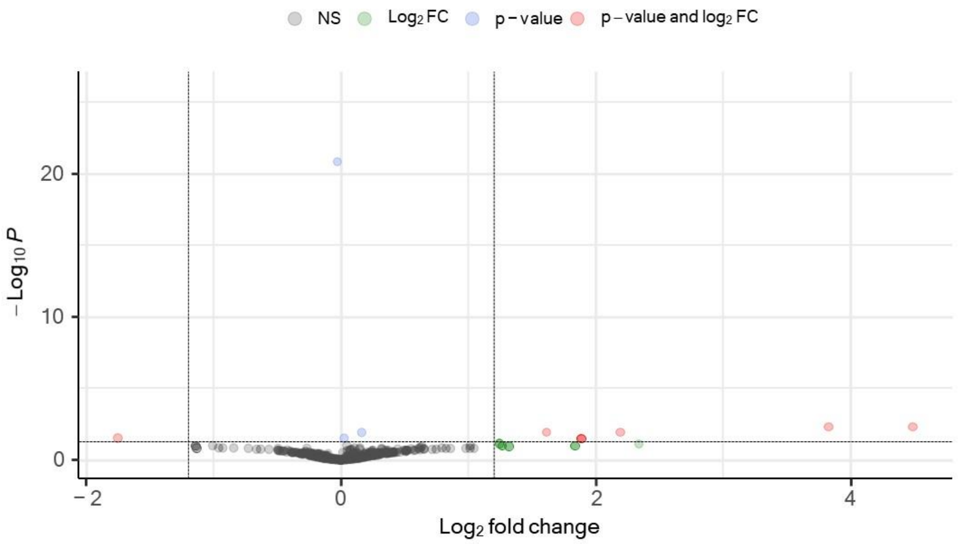

Supplementary Figure S1. Volcano plot of differentially regulated baseline plasma circulating microRNAs in baseline microRNA patterns in hospitalized SARS-CoV-2 acute infected patients. Differentially expressed (DE) miRNAs were determined by comparing plasma miRNA expression profiles between **A)** SARS-CoV-2 patients requiring high-flow or low-flow oxygen support and **B)** SARS-CoV-2 patients requiring high-flow oxygen or no oxygen support. To

identify biomarker candidates, cutoffs of effect size (fold change [FC] >1.2) and significance level (adjusted p [padj] <0.05) were used. Sets of 31 and 6 DE (red) miRNAs were identified, respectively.
