# Supplementary material for: Altered Plasma microRNA Signature in Hospitalized COVID-19 Patients Requiring Oxygen Support
Source: Microorganisms. 2024 Feb 21;12(3):440. doi: 10.3390/microorganisms12030440 (PMC10972147; doi:10.3390/microorganisms12030440)
Supplement: Supplementary file 1 [file microorganisms-12-00440-s001.zip › Supplementary Figure S2.pdf]

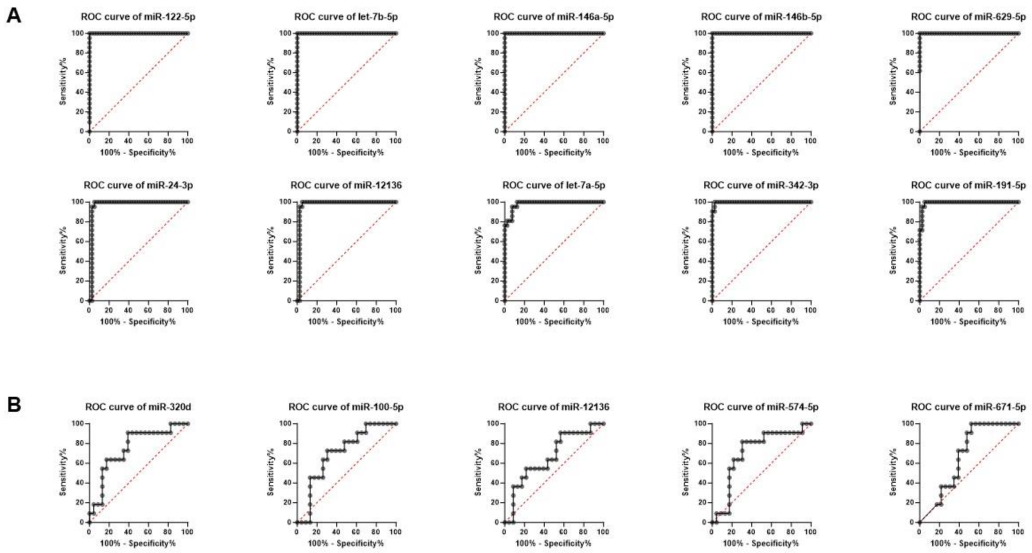

Supplementary Figure S2. ROC curves show the diagnostic performance of study miRNAs. A) ROC curves of the top 10 DE miRNAs (miR-122-5p, let-7b-5p, miR-146a-5p, miR-146b-5p, miR-629-5p, miR-24-3p, miR-12136, let-7a-5p, miR-342-3p, and miR-191-5p) between SARS-CoV-2–infected patients versus uninfected healthy individuals and **B)** ROC curves of the top 5 DE miRNAs (miR-320d, miR-100-5p, miR-12136, miR-574-5p and miR-671-5p) between SARS-CoV-2–infected patients requiring high-flow versus low-flow oxygen support.
