# Supplementary material for: Altered Plasma microRNA Signature in Hospitalized COVID-19 Patients Requiring Oxygen Support
Source: Microorganisms. 2024 Feb 21;12(3):440. doi: 10.3390/microorganisms12030440 (PMC10972147; doi:10.3390/microorganisms12030440)
Supplement: Supplementary file 1 [file microorganisms-12-00440-s001.zip › Supplementary Table S1.pdf]

Supplementary Table S1. Differential expression at the total miRNA level for SARS-CoV-2–infected patients versus uninfected healthy individuals.

miRNA	Base mean[a]	Fold Change[b]	p adjusted[c]
hsa-miR-122-5p	1313.30	95.01	2.51E-32
hsa-let-7b-5p	12193.88	14.82	6.46E-32
hsa-miR-146a-5p	16302.81	11.79	5.50E-26
hsa-miR-342-3p	1399.90	-10.85	9.50E-26
hsa-miR-146b-5p	1994.97	21.11	1.83E-25
hsa-miR-629-5p	82.34	153.28	3.76E-25
hsa-miR-24-3p	567.24	11.71	1.11E-24
hsa-miR-12136	429.31	48.84	4.14E-23
hsa-let-7a-5p	29587.51	10.93	4.65E-22
hsa-miR-191-5p	303583.51	-4.75	2.21E-21
hsa-let-7c-5p	387.92	10.27	2.45E-20
hsa-miR-1260b	309.64	6.50	2.68E-19
hsa-let-7e-5p	3253.50	10.41	1.73E-17
hsa-miR-23a-5p	51.87	120.26	4.53E-17
hsa-miR-223-5p	344.40	4.35	4.40E-15
hsa-miR-6804-5p	23.44	304.44	1.33E-14
hsa-miR-574-5p	158.46	20.11	1.73E-14
hsa-miR-98-5p	2116.63	6.77	7.70E-14
hsa-miR-6837-3p	90.14	-5.74	7.98E-14
hsa-miR-1908-3p	139.86	22.32	1.02E-13
hsa-let-7g-5p	5961.40	5.10	2.03E-13
hsa-miR-30b-5p	2098.68	-5.54	2.66E-13
hsa-miR-589-5p	593.33	3.36	1.37E-12
hsa-miR-150-5p	3699.99	-7.31	3.39E-12
hsa-miR-328-3p	976.41	4.96	3.70E-12
hsa-miR-26b-3p	728.45	-4.23	9.72E-12
hsa-miR-181a-5p	62830.61	-3.16	3.59E-11
hsa-miR-182-5p	2886.55	-3.46	1.24E-10
hsa-miR-188-5p	33.34	-16.68	2.56E-10
hsa-miR-145-5p	111.63	-6.28	3.38E-10
hsa-miR-574-3p	264.67	3.20	3.38E-10
hsa-miR-130b-5p	327.56	3.73	4.68E-10
hsa-miR-142-3p	818.85	-4.47	6.70E-10
hsa-let-7f-5p	81822.16	3.94	9.01E-10
hsa-miR-199b-3p	14190.56	2.48	9.69E-10
hsa-miR-10b-5p	28621.20	-4.76	5.96E-09
hsa-miR-577	20.33	-15.35	7.16E-09
hsa-miR-548e-3p	113.04	2.71	3.00E-08
hsa-miR-7849-3p	109.44	-3.51	5.30E-08
hsa-miR-186-3p	12.61	-17.75	6.06E-08
hsa-miR-193b-5p	97.93	19.70	6.06E-08
hsa-miR-4433b-5p	820.07	3.63	6.06E-08

hsa-miR-193a-3p	5.33	-34.54	7.77E-08
hsa-miR-128-3p	4061.79	2.17	9.21E-08
hsa-miR-4741	23.45	20.11	1.12E-07
hsa-miR-21-3p	7034.52	-2.43	1.15E-07
hsa-miR-193a-5p	10.76	67.18	1.19E-07
hsa-miR-10399-5p	244.03	3.32	3.65E-07
hsa-miR-654-5p	115.83	5.66	4.30E-07
hsa-miR-181c-5p	1485.90	-2.87	5.12E-07
hsa-miR-877-5p	2437.74	-4.26	1.71E-06
hsa-miR-628-3p	309.27	-3.41	2.26E-06
hsa-miR-423-3p	30851.04	3.18	2.93E-06
hsa-miR-1285-3p	460.89	-3.16	3.66E-06
hsa-miR-3173-5p	13.06	6.19	5.62E-06
hsa-miR-381-3p	1182.08	-4.56	5.62E-06
hsa-miR-3168	72.72	103.25	6.85E-06
hsa-miR-148b-3p	5441.18	-1.96	7.32E-06
hsa-miR-1908-5p	44.88	5.90	8.57E-06
hsa-miR-885-5p	20.46	13.18	9.56E-06
hsa-miR-4657	12.50	23.43	1.25E-05
hsa-miR-10a-5p	24773.78	-2.58	1.26E-05
hsa-miR-186-5p	26793.94	-1.74	1.65E-05
hsa-miR-4531	26.64	2.48	1.65E-05
hsa-miR-134-5p	748.81	5.78	1.67E-05
hsa-miR-1294	7.58	13.83	1.87E-05
hsa-miR-494-3p	46.36	-4.08	1.93E-05
hsa-let-7i-5p	47729.96	2.89	2.09E-05
hsa-miR-6763-5p	22.80	1.75	2.11E-05
hsa-miR-376a-5p	38.02	-5.78	2.49E-05
hsa-miR-411-3p	17.81	-4.00	2.89E-05
hsa-let-7d-3p	3545.22	2.28	3.04E-05
hsa-miR-3173-3p	4.44	17.88	3.84E-05
hsa-miR-6842-3p	225.38	-2.91	3.84E-05
hsa-let-7d-5p	4120.77	2.16	5.69E-05
hsa-miR-874-3p	78.00	-2.85	6.44E-05
hsa-miR-28-5p	1378.73	1.60	7.36E-05
hsa-miR-582-3p	14.01	-4.92	7.90E-05
hsa-miR-1255b-5p	9.38	12.21	8.58E-05
hsa-miR-483-5p	128.56	13.55	9.48E-05
hsa-miR-484	3913.69	1.65	0.00011099
hsa-miR-486-3p	1107.54	2.43	0.00014081
hsa-miR-224-5p	192.51	-3.25	0.00020053
hsa-miR-7110-5p	16.49	1.33	0.00035199
hsa-miR-5683	31.12	-4.63	0.0004265
hsa-miR-148b-5p	103.76	-2.57	0.00048516
hsa-miR-199a-5p	1231.29	-1.79	0.00049544
hsa-miR-4687-3p	18.44	1.85	0.00053459

hsa-miR-15a-3p	2.29	-18.51	0.00055438
hsa-miR-548ax	27.45	-2.23	0.00061659
hsa-miR-4746-5p	53.97	-2.10	0.00070581
hsa-miR-486-5p	1209535.88	-2.19	0.00072654
hsa-miR-103a-2-5p	16.68	-5.31	0.00073059
hsa-miR-3677-3p	19.59	-7.62	0.00079776
hsa-miR-152-5p	12.82	-3.84	0.00083844
hsa-miR-26a-5p	119270.43	-1.96	0.00084999
hsa-miR-144-3p	760.43	-2.71	0.00093239
hsa-miR-125a-3p	18.95	4.44	0.00094233
hsa-miR-758-3p	47.81	-2.99	0.00094233
hsa-miR-2467-5p	13.06	-3.48	0.00104643
hsa-miR-497-5p	99.39	-2.50	0.00104924
hsa-miR-615-3p	3.13	-19.16	0.0012779
hsa-miR-937-3p	12.11	3.34	0.00138689
hsa-miR-101-5p	10.99	-4.29	0.00147975
hsa-miR-127-5p	19.91	-5.62	0.00147975
hsa-miR-1306-5p	13.30	3.10	0.00148194
hsa-miR-320d	353.67	4.26	0.00149759
hsa-miR-183-5p	95.95	-3.36	0.00157936
hsa-miR-6511b-3p	29.59	3.65	0.00161186
hsa-miR-22-3p	537575.27	2.03	0.0017982
hsa-miR-10b-3p	4.64	-6.36	0.00186359
hsa-miR-4786-5p	7.93	-4.32	0.00240482
hsa-miR-500a-3p	534.36	-2.07	0.00255457
hsa-miR-1275	42.17	4.69	0.00289855
hsa-miR-1307-5p	2755.93	-2.13	0.00293017
hsa-miR-1296-5p	31.29	-2.57	0.00344013
hsa-miR-125a-5p	4522.33	-2.06	0.00349616
hsa-miR-190a-5p	14.06	-3.43	0.00350676
hsa-miR-1285-5p	4.39	7.78	0.00370909
hsa-miR-323a-3p	91.61	-2.50	0.00406705
hsa-miR-625-3p	236.66	2.69	0.00421319
hsa-miR-345-5p	951.84	1.69	0.00461837
hsa-miR-589-3p	36.39	-2.48	0.00462275
hsa-miR-887-3p	3.13	-7.41	0.00462275
hsa-miR-361-3p	190.26	1.85	0.00469694
hsa-miR-6843-3p	10.38	3.46	0.00483652
hsa-miR-185-5p	42.16	3.07	0.00487442
hsa-miR-33a-5p	59.81	-2.10	0.00487442
hsa-miR-369-3p	164.54	-2.50	0.00505921
hsa-miR-425-3p	116.73	-1.69	0.00556425
hsa-miR-136-5p	72.47	-2.33	0.00698101
hsa-miR-1468-5p	317.95	-1.79	0.00717601
hsa-miR-542-3p	9.26	3.32	0.00729782
hsa-miR-203a-3p	9.12	3.43	0.00760719

hsa-miR-6810-5p	4.04	-6.19	0.00892644
hsa-miR-128-1-5p	56.33	-1.97	0.00951947
hsa-miR-548o-5p	44.69	-1.96	0.00951947
hsa-miR-370-3p	279.02	-2.75	0.01091894
hsa-miR-423-5p	466133.15	2.71	0.01091894
hsa-miR-576-5p	64.95	2.19	0.01125822
hsa-miR-889-3p	320.09	-2.55	0.01171197
hsa-miR-487b-3p	208.88	-2.07	0.01205707
hsa-miR-6515-5p	36.01	2.69	0.01205707
hsa-let-7f-1-3p	21.82	2.53	0.01451649
hsa-miR-584-3p	3.74	-3.55	0.01494697
hsa-miR-342-5p	136.99	2.11	0.01571134
hsa-miR-24-2-5p	46.22	-1.91	0.01602991
hsa-miR-138-5p	7.79	-3.71	0.01632269
hsa-miR-33a-3p	10.08	-2.93	0.01634623
hsa-miR-1292-5p	8.32	-3.63	0.01786217
hsa-miR-375-3p	1074.38	-2.22	0.01788263
hsa-miR-410-3p	1795.83	-2.13	0.01875577
hsa-miR-4448	16.70	4.53	0.02015719
hsa-miR-4775	9.98	-2.75	0.02016087
hsa-miR-545-3p	1.61	-15.24	0.02078413
hsa-miR-6832-5p	4.35	-4.08	0.02308529
hsa-miR-30a-5p	3523.69	1.73	0.02346137
hsa-miR-197-3p	266.75	1.57	0.02464744
hsa-miR-331-3p	131.30	-2.31	0.02618439
hsa-miR-4435	124.86	-1.77	0.026672
hsa-miR-452-5p	30.82	2.79	0.02796616
hsa-miR-338-3p	144.10	-1.71	0.02885367
hsa-miR-760	12.41	2.55	0.02900832
hsa-miR-151a-3p	71062.92	-1.28	0.03094333
hsa-miR-369-5p	139.89	-2.11	0.03295771
hsa-miR-4645-3p	14.94	-1.99	0.03344232
hsa-miR-6777-3p	5.01	3.39	0.03416958
hsa-miR-200a-3p	11.23	-2.66	0.03428082
hsa-miR-664b-5p	2.36	47.50	0.03428082
hsa-miR-5189-3p	30.96	-2.62	0.03466647
hsa-miR-11400	497.23	-1.65	0.03530447
hsa-miR-7976	32.55	2.64	0.03624748
hsa-miR-431-3p	35.58	-2.03	0.03768563
hsa-miR-20a-3p	3.92	-2.97	0.03789382
hsa-miR-6891-5p	3.14	2.25	0.03789382
hsa-miR-299-3p	18.93	-2.43	0.0388085
hsa-miR-196b-5p	76.69	2.08	0.03908651
hsa-miR-30e-3p	1538.75	-1.32	0.04042597
hsa-miR-3158-3p	115.69	-2.38	0.04084722
hsa-miR-320a-3p	28910.15	2.30	0.04316291

hsa-miR-34a-5p	2.55	2.13	0.04341515
hsa-miR-450b-5p	67.36	1.97	0.0453496
hsa-miR-130a-3p	5512.58	-1.49	0.04656576
hsa-miR-154-5p	6.22	-2.87	0.04807832
hsa-miR-4454	20.25	-1.83	0.04810743
hsa-miR-411-5p	686.10	-2.00	0.04892527
hsa-miR-6818-5p	6.11	-2.17	0.04975826
hsa-miR-532-5p	1095.76	-1.58	0.04979005
hsa-miR-548l	3.49	-2.75	0.04979045
hsa-miR-6862-5p	15.01	2.91	0.04979045

[a]The average of the normalized count values according to DeSeq2.
[b]The effect size estimate according to DeSeq2.
[c]P value adjusted for false discovery rate by the method of Benjamini and Hochberg.
