# Supplementary material for: Altered Plasma microRNA Signature in Hospitalized COVID-19 Patients Requiring Oxygen Support
Source: Microorganisms. 2024 Feb 21;12(3):440. doi: 10.3390/microorganisms12030440 (PMC10972147; doi:10.3390/microorganisms12030440)
Supplement: Supplementary file 1 [file microorganisms-12-00440-s001.zip › Supplementary Table S2.pdf]

Supplementary Table S2. Differential expression at the total miRNA level between SARS-CoV-2–infected patients requiring high-flow versus low-flow oxygen support.

miRNA	Base mean[a]	Fold Change[b]	p adjusted[c]
hsa-miR-320d	364.54	4.79	0.00255151
hsa-miR-100-5p	277.51	2.31	0.00918043
hsa-miR-12136	429.43	2.99	0.00918043
hsa-miR-574-5p	158.823	2.93	0.00918043
hsa-miR-671-5p	75.573	7.78	0.00918043
hsa-miR-1307-3p	1713.60	2.69	0.0132755
hsa-miR-127-3p	33515.55	2.50	0.01774758
hsa-miR-1275	42.775	3.76	0.01774758
hsa-miR-1908-3p	145.16	2.66	0.01774758
hsa-miR-30c-1-3p	192.68	1.96	0.01774758
hsa-miR-320a-3p	28912.58	2.50	0.01774758
hsa-miR-331-3p	131.21	2.58	0.01774758
hsa-miR-337-5p	7.71	2.75	0.01774758
hsa-miR-378a-3p	10825.97	2.81	0.01774758
hsa-miR-378d	443.14	2.89	0.01774758
hsa-miR-185-3p	249.53	2.04	0.02343964
hsa-let-7i-5p	47716.11	1.75	0.02722205
hsa-miR-191-3p	302.38	1.59	0.03204814
hsa-miR-193b-5p	97.87	6.36	0.03204814
hsa-miR-423-3p	30856.677	1.73	0.03329827
hsa-miR-339-5p	446.317	1.74	0.03513266
hsa-miR-17-3p	369.33	1.99	0.03601704
hsa-miR-320b	113.88	2.07	0.03706333
hsa-miR-134-5p	748.97	2.17	0.03900094
hsa-miR-1273h-5p	76.97	2.17	0.04065254
hsa-miR-7848-3p	9.86	2.17	0.0407411
hsa-miR-423-5p	466152.26	2.01	0.04158325
hsa-miR-582-3p	13.676	-2.31	0.0432867
hsa-miR-561-5p	5.856	-2.57	0.04918054
hsa-miR-144-5p	603.74	-1.89	0.04932173
hsa-miR-181a-2-3p	522.59	1.41	0.04932173

[a]The average of the normalized count values according to DeSeq2.
[b]The effect size estimate according to DeSeq2.
[c]P value adjusted for false discovery rate by the method of Benjamini and Hochberg.
