# Supplementary material for: Altered Plasma microRNA Signature in Hospitalized COVID-19 Patients Requiring Oxygen Support
Source: Microorganisms. 2024 Feb 21;12(3):440. doi: 10.3390/microorganisms12030440 (PMC10972147; doi:10.3390/microorganisms12030440)
Supplement: Supplementary file 1 [file microorganisms-12-00440-s001.zip › Supplementary Table S3.pdf]

Supplementary Table S3 Differential expression at the total miRNA level between SARS-CoV-2–infected patients requiring high-flow versus no oxygen support.

miRNA	Base mean[a]	Fold Change[b]	p adjusted[c]
hsa-miR-671-5p	75.57	22.47	0.00519451
hsa-miR-6819-5p	11.13	14.22	0.00420542
hsa-miR-100-5p	277.51	3.05	0.01181046
hsa-miR-1306-3p	18.82	4.56	0.01227122
hsa-miR-3688-3p	20.97	-3.36	0.03054533
hsa-miR-4646-5p	12.31	3.71	0.03846312

[a]The average of the normalized count values according to DeSeq2.
[b]The effect size estimate according to DeSeq2.
[c]P value adjusted for false discovery rate by the method of Benjamini and Hochberg.
